# Dynamic Modelling and Control Strategy Analysis of a Lower-Limb Exoskeleton

**DOI:** 10.3390/s26072124

**Published:** 2026-03-29

**Authors:** Huanrong Xiao, Teng Ran, Afang Jin

**Affiliations:** College of Mechanical Engineering, Xinjiang University, Ürümqi 830047, China; 107552301381@stu.xju.edu.cn (H.X.); ranteng@xju.edu.cn (T.R.)

**Keywords:** lower-limb exoskeleton, Lagrangian equation, dynamics modelling, computed torque method, trajectory tracking control

## Abstract

Lower-limb exoskeleton robots play a pivotal role in rehabilitation medicine and assistive augmentation, where precise dynamic modelling and trajectory tracking control are fundamental to effective assistance. Existing models predominantly focus on hip and knee rotational degrees of freedom, with insufficient attention to ankle dynamics and pelvic translation. To address these limitations, this paper establishes a sagittal-plane dynamic model comprising nine generalised coordinates, treating the human lower limb and exoskeleton as an integrated coupled system. A seven-segment kinematic model encompassing the trunk, bilateral thighs, shanks, and feet is constructed via a modified Denavit–Hartenberg parameter method, and dynamic equations are derived using Lagrangian formulation. Three control strategies—PD control, PD with gravity compensation, and the computed torque method—are designed and evaluated through simulations using gait data from five subjects (two self-collected, three from a public dataset) acquired via Vicon motion capture. Results demonstrate that the computed torque method achieves a joint angle tracking root mean square error (RMSE) of 0.59°, representing an 86.3% improvement over conventional PD control, while maintaining a low control torque RMS of 4.44 N·m. The controller exhibits stable tracking performance across walking speeds of 0.4–1.45 m/s, validating the effectiveness of the proposed model and control strategies.

## 1. Introduction

Lower-limb exoskeleton robots are wearable human–machine integrated intelligent devices capable of providing wearers with movement assistance or enhanced physical capabilities. With the intensification of population ageing and the growing demand for rehabilitation healthcare, lower-limb exoskeletons have demonstrated broad application prospects in medical rehabilitation, elderly and disability assistance, and military load-bearing applications [1,2]. Precise dynamic modelling forms the foundation for achieving high-performance exoskeleton control, directly influencing the system’s assistive efficacy and human–machine interaction comfort [3].

Regarding lower-limb exoskeleton dynamic modelling, researchers have employed models of varying complexity. Early studies predominantly utilised low-degree-of-freedom (DOF) models, focusing primarily on hip and knee joint rotations. Chen et al. [4] established a 2-DOF dynamic model based on Lagrange equations and achieved human–robot collaborative control. Within a similar framework, Wu et al. [5] incorporated parameter identification and adaptive backstepping control to enhance adaptability to parameter uncertainties. Shi et al. [6] developed a 2-DOF hip-knee dynamic model based on the Lagrangian method and, combined with parameter identification, designed a backstepping controller to achieve trajectory tracking control of a lower-limb exoskeleton. In recent years, researchers have progressively adopted higher-DOF models to accurately describe gait dynamics. Yan et al. [7] developed a 4-DOF swinging dynamics model for lower-limb exoskeletons, incorporating human–machine coupling effects. Yan et al. [8] further explicitly introduced human–machine coupling interaction forces into the dynamic modelling, constructing a coupled multi-rigid-body system of the human and exoskeleton, and analysed the coupled dynamic characteristics through experimental calibration of interaction parameters. Long et al. [9] developed an electrically actuated lower-limb assistive exoskeleton and evaluated the torque requirements at each joint during assisted walking through dynamic analysis, providing a reference for subsequent high-DOF model design. However, existing models still commonly employ simplifying assumptions. Many studies focus solely on the rotational degrees of freedom of the hip and knee joints, neglecting the ankle joint’s crucial contribution to gait stability and propulsive force generation [10] and failing to adequately account for the influence of pelvic translation on centre-of-gravity control and system dynamics [11,12].

Regarding lower-limb exoskeleton control strategies, researchers have proposed multiple approaches. PD control, favoured for its structural simplicity and ease of implementation, was widely employed in early studies [13]; however, its failure to account for system dynamics led to degraded tracking performance during rapid movements or load changes. To enhance tracking accuracy, researchers introduced gravity compensation strategies, reducing steady-state error by compensating for the system’s gravitational term [14]. In recent years, control methods based on precise dynamic models have emerged as a research focus. Among these, the computed torque method (CTM) has gained widespread application due to its ability to effectively compensate for system nonlinear dynamic characteristics, theoretically enabling asymptotically stable trajectory tracking [15,16]. Yu et al. [17] employed the computed torque method to achieve gait control in an active lower-limb exoskeleton and validated trajectory tracking performance under human–machine interaction disturbances. Zhang et al. [18] developed a hip exoskeleton with balance capacities and achieved compliant human–machine interaction through admittance-based control. [19] proposed a neural network-based robust computed torque control method, utilising RBF neural networks to compensate for modelling errors, thereby improving system robustness and tracking accuracy. Nevertheless, existing research remains insufficient in quantitatively analysing the relationship between dynamic model accuracy and control performance, particularly regarding the co-optimisation of control accuracy and energy consumption, where relevant studies are limited [20]. Gao et al. [21] investigated the human–machine coupling dynamics and assistance performance of an ankle exoskeleton, providing further insight into the dynamic contribution of the ankle joint during assisted gait.

**Figure 1:**
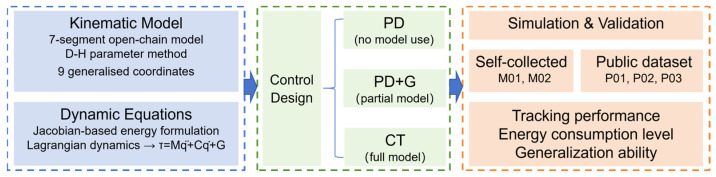
Flowchart of the overall research workflow.

To address these issues, this paper undertakes the following research contributions: (1) Establishment of a 9-DOF dynamic model: treating the human lower limb and exoskeleton as an integrated rigid-body system, a dynamic model comprising nine generalised coordinates (seven joint angles and two pelvic translation components) within the sagittal plane is developed based on Lagrange equations. Compared to existing 2–4 DOF models, this approach simultaneously accounts for hip, knee, and ankle joint rotations as well as pelvic translation, enabling a more comprehensive description of the dynamic characteristics during gait motion. (2) Comparative study based on existing control methods: three control strategies—PD control, PD with gravity compensation (PD + G), and the computed torque method (CT)—are designed and subjected to systematic comparative analysis from the perspectives of tracking accuracy and energy consumption. (3) Multi-speed, multi-subject gait data validation: based on multi-speed gait data collected from two healthy subjects via a Vicon motion capture system, and supplemented by data from three subjects in the public gait dataset established by Rosenberg et al. [22] at walking speeds of 1.30–1.45 m/s, the accuracy of the dynamic model and the tracking performance and generalisation capability of the control strategies across different walking speeds and individuals are validated from both self-collected and publicly available dataset perspectives. To facilitate understanding of the overall research procedure and the connections among the main sections, a workflow of the present study is provided in Figure 1.

## 2. Materials and Methods

### 2.1. Lower Limb Exoskeleton System and Kinematic Model

The lower-limb exoskeleton forms a tightly coupled human–machine system with the wearer during motion. Under normal walking conditions, human joint movements primarily occur within the sagittal plane, with relatively minor motion in the coronal and transverse planes. Based on these characteristics, a two-dimensional rigid-body dynamic model is established within the sagittal plane. Each exoskeleton component and its corresponding human body segment are treated as tightly bound equivalent rigid units with no relative motion between them, and the assistive effect of the exoskeleton is simplified as a unidirectional ideal torque input at each joint. The equivalent geometric length of each segment is assumed equal to the anatomical length of the corresponding human limb segment, and each segment is modelled as a uniform rigid rod with its centre of mass located at the geometric midpoint. Under these assumptions, the human–machine coupled system is represented as a seven-bar open-chain serial mechanism within the sagittal plane. Segment 1 represents the trunk; segments 2–4 correspond to the left thigh, left shank, and left foot; and segments 5–7 correspond to the right thigh, right shank, and right foot.

**Figure 2:**
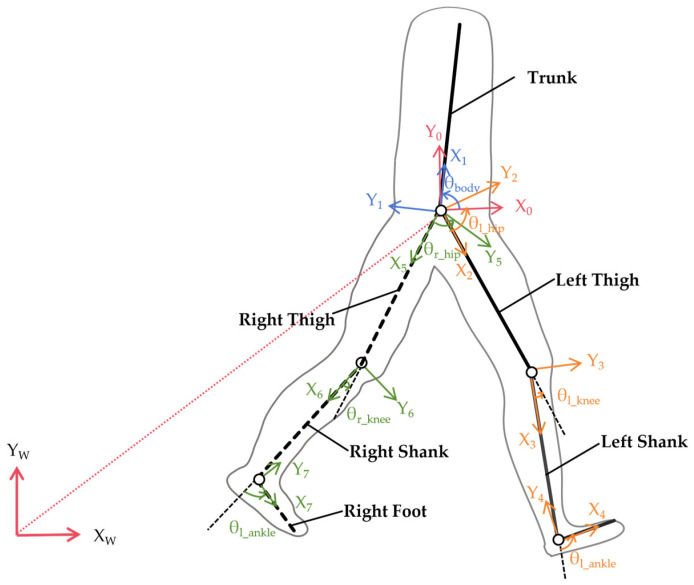
Definition of the coordinate system for the lower-limb exoskeleton system.

Coordinate systems are established following the Denavit–Hartenberg (D-H) method. An inertial reference frame {W} is fixed to the ground, with a floating base frame {0} defined at the pelvis reference point. Pelvic translation in the horizontal and vertical directions is described by x_0_ and y_0_, respectively. Each segment coordinate system {i} (i = 1, 2, …, 7) is established with the proximal joint as its origin, the *Z*-axis perpendicular to the sagittal plane pointing outward, and the *X*-axis aligned with the segment’s longitudinal axis pointing distally. The coordinate system definitions are illustrated in Figure 2, and the corresponding D-H parameters are listed in Table 1. In Figure 2, different colors are used to distinguish coordinate systems for clarity: red indicates the inertial frame {W} and pelvic frame {0}, blue denotes the trunk coordinate system {1}, and orange and green represent the left and right lower-limb kinematic chains ({2–4} and {5–7}), respectively. The conventions adopted are: θ_1_ = θ_body_, θ_2_ = θ_l_hip_, θ_3_ = θ_l_knee_, θ_4_ = θ_l_ankle_, θ_5_ = θ_r_hip_, θ_6_ = θ_r_knee_, and θ_7_ = θ_r_ankle_. The parameters l_l_thigh_ = L_T_, l_l_shank_ = L_S_, and l_l_foot_ = L_F_ denote the left thigh, shank, and foot lengths, while l_r_thigh_ = R_T_, l_r_shank_ = R_S_, and l_r_foot_ = R_F_ denote the right thigh, shank, and foot lengths.

Since all joint axes are parallel to the Z_W_ axis, the standard D-H transformation matrix reduces to a pure planar rotation-plus-translation form. The homogeneous transformation matrix from frame {i − 1} to frame {i} is simplified as follows:

(1) Tii−1=cosθi−sinθi0ai−1sinθicosθi0000100001

The transformation matrix of the pelvic base frame {0} relative to the world inertial frame {W} is determined by the translational coordinates x_0_ and y_0_:

(2) T0W=100x0010y000100001

By sequentially substituting the geometric parameters a_i−1_ into the general transformation matrix, the specific local transformation matrices for the left lower limb joints can be explicitly derived. Specifically, for the hip, knee, and ankle joints, the matrices are:

(3) T20=cosθ2−sinθ200sinθ2cosθ20000100001, T32=cosθ3−sinθ30LTsinθ3cosθ30000100001, T43=cosθ4−sinθ40LSsinθ4cosθ40000100001

The global position and orientation of each segment are then obtained through successive matrix multiplication. For instance, the transformation matrices for the left knee and left ankle relative to the world frame are derived recursively as T3W = T0W·T20·T32 and T4W = T3W·T3. The right leg chain follows an identical recursive procedure. Under the uniform rigid rod assumption, letting l_i_ denote the generic geometric length of segment i (e.g., l_2_ = L_T_, l_3_ = L_S_, etc.), its centre of mass in the world frame can be uniformly expressed as follows:

(4) rc(i) = TiW·li2, 0, 0, 1T

### 2.2. Lower Limb Exoskeleton Dynamic Model

#### 2.2.1. Generalised Coordinates and Kinematic Jacobian

Building upon the kinematic model, the dynamic equations of the human–machine coupled system are formulated using the Lagrange method based on Jacobian matrices. Initially, the system configuration is described by a vector containing the seven joint angles and the two pelvic translation components:

(5) $$q={\left[\theta_{1},\;\theta_{2},\;\theta_{3},\;\theta_{4},\;\theta_{5},\;\theta_{6},\;\theta_{7},\;x_{0},\;y_{0}\right]}^{T}\in {\mathbb{R}}^{9\times 1}$$

Since the control objective of this study focuses on the six active lower-limb joints, the trunk rotation angle θ_1_ is treated as a passive degree of freedom and is assumed to remain fixed at the vertical posture (θ_1_ = 90°). No active drive torque is applied to θ_1_, x_0_, or y_0_. This assumption reduces the control dimensionality while strictly preserving the dynamic coupling effects of pelvic translation on lower-limb joint dynamics.

To formulate the kinetic energy, the absolute linear and angular velocities of each segment’s centre of mass must be mapped from the joint velocity vector $\dot{q}$. Differentiating the position vector $r_{c}^{\left(i\right)}(q)$ with respect to time yields the linear velocity:

(6) $${\dot{r}}_{c}^{\left(i\right)}=\frac{\partial r_{c}^{\left(i\right)}}{\partial q}\dot{q}=J_{v}^{\left(i\right)}(q)\dot{q}$$

where $J_{v}^{\left(i\right)}(q)\in {\mathbb{R}}^{2\times 9}$ is the linear velocity Jacobian matrix of segment i. Due to the serial open-chain topology, only the generalised coordinates corresponding to joints upstream of segment i contribute non-zero columns to $J_{v}^{\left(i\right)}$.

Similarly, for planar motion, the absolute angular velocity of segment i about the sagittal normal axis is the algebraic sum of all preceding joint angular velocities. This relationship is written compactly as $\omega^{\left(i\right)}=J_{\omega }^{\left(i\right)}\dot{q}$, where $J_{\omega }^{\left(i\right)}\in {\mathbb{R}}^{1\times 9}$ is the angular velocity Jacobian. For example, the angular velocity Jacobian of the left shank (segment 3) is explicitly given by $J_{\omega }^{\left(3\right)}=[0,1,1,0,0,0,0,0,0]$. Notably, pelvic translations (x_0_, y_0_) do not generate rotational motion; hence, the final two elements of every $J_{\omega }^{\left(i\right)}$ are strictly zero.

#### 2.2.2. System Energies and Dynamic Equations

The kinetic energy of any segment i (i = 1, 2, …, 7) is the sum of its translational and rotational kinetic energies:

(7) $$K_{i}=\frac{1}{2}m_{i}{\left\|J_{v}^{\left(i\right)}\dot{q}\right\|}^{2}+\frac{1}{2}I_{i}{\left(J_{\omega }^{\left(i\right)}\dot{q}\right)}^{2}=\frac{1}{2}{\dot{q}}^{T}\left[m_{i}J_{v}^{(i)T}J_{v}^{\left(i\right)}+I_{i}J_{\omega }^{(i)T}J_{\omega }^{\left(i\right)}\right]\dot{q}$$

where m_i_ is the total equivalent mass of segment i, and $I_{i}=\frac{1}{12}m_{i}l_{i}^{2}$ is the moment of inertia about the centre of mass. Summing the kinetic energy across all seven segments yields the total system kinetic energy $K=\sum_{i=1}^{7}K_{i}=\frac{1}{2}{\dot{q}}^{T}M(q)\dot{q}$. Here, the symmetric and positive definite generalised mass matrix $M(q)\in {\mathbb{R}}^{9\times 9}$ is defined as follows:

(8) M(q)=∑i=17miJv(i)(Tq)Jv(i)(q)+IiJω(i)TJω(i)

The gravitational potential energy of the system is the sum of the potential energies of all segments, formulated as $U(q)=\sum_{i=1}^{7}m_{i}gy_{c}^{\left(i\right)}(q)$, where $g=9.81\;{m/s}^{2}$ and $y_{c}^{\left(i\right)}$ is the vertical coordinate of the respective centre of mass.

By constructing the Lagrangian L=K−U and applying Lagrange’s equations to each degree of freedom, the system dynamics can be derived. To unify the subscript notation and facilitate the final matrix formulation, the translational coordinates are redefined as angular-style indices by defining θ_8_ = x_0_ and θ_9_ = y_0_. The generalised coordinate vector can then be rewritten as $q={\left[\theta_{1},\theta_{2},\ldots ,\theta_{9}\right]}^{T}$. Substituting L into the standard Euler-Lagrange equation yields the final matrix-form dynamic equation:

(9) $$M(q)\ddot{q}+C(q,\dot{q})\dot{q}+G(q)=\tau$$

The Coriolis and centrifugal force matrix $C(q,\dot{q})\in {\mathbb{R}}^{9\times 9}$ is derived using the Christoffel symbols of the first kind associated with the mass matrix, which quantify how changes in system configuration alter the effective inertia:

(10) $$C_{kj}=\sum_{l=1}^{9}\Gamma_{kjl}{\dot{q}}_{l},\;\Gamma_{kjl}=\frac{1}{2}\;\left(\frac{\partial M_{kj}}{\partial q_{l}}+\frac{\partial M_{kl}}{\partial q_{j}}-\frac{\partial M_{jl}}{\partial q_{k}}\right)$$

The gravity vector $G(q)\in {\mathbb{R}}^{9\times 1}$ captures the configuration-dependent gravitational loads, with its elements given by the following:

(11) $$G_{k}(q)=\frac{\partial U}{\partial q_{k}}=\sum_{i=1}^{7}m_{i}g\frac{\partial y_{c}^{\left(i\right)}}{\partial q_{k}}$$

The generalised force vector τ comprises the actuation torques provided to the joints.

An essential mathematical property of this Lagrangian formulation is that the matrix $\left(\dot{M}-2C\right)$ is skew-symmetric. This property plays a pivotal role in designing the Lyapunov function and proving the asymptotic stability of the computed torque method presented in Section 2.3.4. The explicit element-wise expressions of M, C, and G are provided in the Appendix A. The MATLAB implementation of the dynamic equations (M, C, and G matrices) is provided as Supplementary Materials.

### 2.3. Control System Design

The core task of the exoskeleton control system is to drive the system to track the desired gait trajectory. Let the desired trajectory be q_d_(t), and the actual trajectory be q(t). The tracking error is defined as $e=q_{d}-q$, and its time derivative is $\dot{e}={\dot{q}}_{d}-\dot{q}$. The three control strategies presented below are formulated within the standard model-based control framework for robotic manipulators [15], and are adapted here for the 9-DOF lower-limb exoskeleton dynamic model established in Section 2.2.

To quantitatively analyse the impact of dynamic modelling accuracy on control performance, three control strategies exhibiting a progressive relationship in their utilisation of the dynamic model are designed: Proportional–Derivative (PD) control, which relies entirely on position and velocity feedback without any dynamic model; PD control with Gravity Compensation (PD + G), which incorporates compensation for the gravitational term G(q) on top of PD control, partially utilising dynamic model information; and the Computed Torque Method (CT), which fully utilises the dynamic model for nonlinear compensation. By comparing the tracking accuracy and energy consumption of these three methods, the accuracy and practical value of the 9-DOF dynamic model established herein can be verified.

It should be noted that in the 9-DOF model of this paper, the actively controlled joints are the bilateral hips, knees, and ankles, comprising six joints in total. The trunk rotation angle and pelvic translation (θ_1_, x_0_, y_0_) serve as passive degrees of freedom, to which no active control torques are applied.

#### 2.3.1. PD Control

PD control is a prevalent feedback control strategy governed by the following control law:

(12) $$\tau (t)=K_{p}e(t)+K_{d}\dot{e}(t)$$

where K_p_ and K_d_ denote the position gain matrix and velocity gain matrix, respectively, both being positive definite diagonal matrices.

PD control features a simple structure and ease of implementation but does not account for the system’s dynamic characteristics. In the presence of gravity and inertial coupling, PD control generates steady-state error and exhibits degraded tracking performance during rapid motions.

#### 2.3.2. PD Control with Gravity Compensation

To mitigate steady-state error induced by gravity, a gravity compensation term is incorporated into the PD control, hereafter referred to as PD + G:

(13) $$\tau =K_{p}e+K_{d}\dot{e}+G(q)$$

The gravity compensation term G (q) counteracts the system’s gravitational influence, effectively reducing steady-state error caused by gravity. However, this approach still neglects the effects of inertial forces and Coriolis forces, limiting its performance during high-speed motion.

#### 2.3.3. Computed Torque Method

The Computed Torque Method (CT) is a control approach based on a precise dynamic model. By compensating for system nonlinearities, it transforms the nonlinear dynamic system into a linearised control problem, thereby achieving higher-precision trajectory tracking.

The control law is designed as follows:

(14) $$\tau =M(q)({\ddot{q}}_{d}+K_{p}e+K_{d}\dot{e})+C(q,\dot{q})+G(q)$$

Here, M(q), $C(q,\dot{q})$, and G(q) respectively denote the mass matrix, Coriolis/centrifugal force vector, and gravity vector derived in Equation (9); ${\ddot{q}}_{d}$ and ${\dot{q}}_{d}$ are the desired joint acceleration and velocity. Substituting the above into the dynamic Equation (9) yields the closed-loop error dynamics:

(15) $$\ddot{e}+K_{d}\dot{e}+K_{p}e=0$$

This constitutes a linear time-invariant system. Selecting K_p_ and K_d_ such that all roots of the characteristic equation s^2^ + K_d_s + K_p_ = 0 possess negative real parts ensures that the tracking error e converges asymptotically to zero.

To achieve critically damped response characteristics while accommodating the dynamic differences between joints, differentiated control gain parameters are specified for different joints. The gain selection satisfies the condition $K_{d}=2\sqrt{K_{p}}$, with specific values given in Table 2.

The three control strategies were selected for comparative analysis based on the following rationale: the three methods form a progressive relationship in their utilisation of the dynamic model. PD control relies entirely on position and velocity feedback without any dynamic model, representing the most fundamental feedback control concept; PD with gravity compensation introduces compensation for the gravity term G(q) on top of PD control, partially utilising dynamic model information; and the computed torque method fully utilises the dynamic model—comprising the mass matrix M, the Coriolis/centrifugal force term C, and the gravity term G—for nonlinear compensation. By comparing the control performance of these three methods, the influence of dynamic modelling accuracy on trajectory tracking control can be quantitatively analysed, thereby validating the accuracy and practical value of the 9-DOF dynamic model constructed herein.

#### 2.3.4. Stability Analysis

To analyse the stability of the computed torque (CT) controller, consider the closed-loop error dynamics obtained by substituting the control law in Equation (14) into the system dynamics in Equation (9). Under the assumption of exact model compensation and perfect state feedback, the closed-loop tracking error system can be written as Equation (15), where *e* = *q*_*d*_ − *q* is the tracking error, $\dot{e}={\dot{q}}_{d}-\dot{q}$ is the tracking error derivative, and K_p_ and K_d_ are positive definite gain matrices.

To prove the asymptotic stability of the equilibrium point (e, ė) = (0, 0), consider the following Lyapunov candidate function:

(16) $$V(e,\dot{e})=\frac{1}{2}{\dot{e}}^{T}\dot{e}+\frac{1}{2}e^{T}K_{p}e$$

Since K_p_ is positive definite, V(e, ė) is positive definite. Taking the time derivative of V along the trajectories of the closed-loop system yields

(17) $$\dot{V}={\dot{e}}^{T}K_{p}e+{\dot{e}}^{T}\ddot{e}$$

Substituting Equation (15) into Equation (17) gives the following:

(18) $$\dot{V}={\dot{e}}^{T}K_{p}e+{\dot{e}}^{T}\left(-K_{d}\dot{e}-K_{p}e\right)=-{\dot{e}}^{T}K_{d}\dot{e}$$

Because K_d_ is positive definite, $\dot{V}=0$ holds only when ė = 0. Combining this condition with the closed-loop error dynamics in Equation (15) further implies e = 0. Therefore, the only invariant set is (e, ė) = (0, 0). According to Lyapunov stability theory and LaSalle’s invariance principle, the equilibrium point of the closed-loop error system is asymptotically stable.

This result indicates that, under ideal model-matching conditions, the computed torque controller can guarantee asymptotic convergence of the joint tracking error to zero.

## 3. Experimental and Simulation Verification

### 3.1. Gait Data Sources and Acquisition Protocol

#### 3.1.1. Experimental Equipment

**Figure 3:**
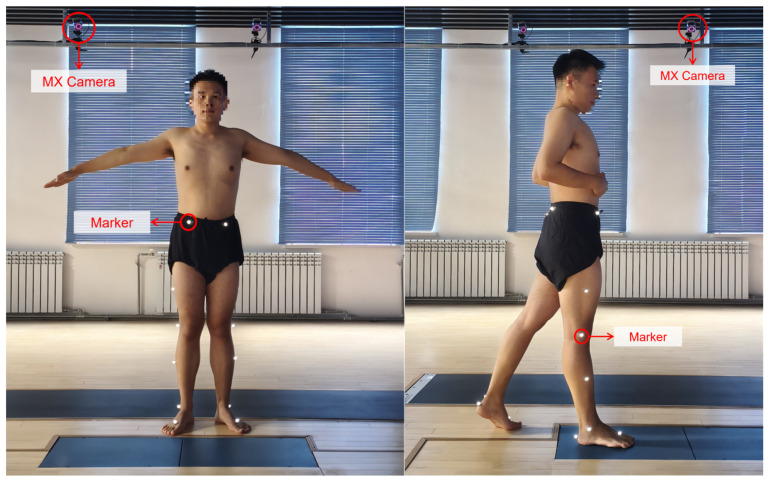
Gait data acquisition experiment.

Gait data acquisition employed the Vicon optical motion capture system (Vicon Motion Systems Ltd., Oxford, UK). The system comprised 12 infrared cameras operating at a sampling frequency of 100 Hz. Human marker points were arranged according to the Plug-in Gait lower-limb model. On each lower limb, reflective markers were placed at the anterior superior iliac spine, posterior superior iliac spine, lateral thigh, lateral knee joint, lateral calf, lateral ankle joint, heel, and second metatarsal head. The scene of lower limb gait collection is shown in Figure 3.

#### 3.1.2. Subjects and Experimental Protocol

To ensure sufficient subject diversity, repeated trials, and speed coverage, this paper constructs the experimental dataset by combining self-collected data with publicly available literature data, yielding a total of five subjects, seven speed–subject conditions, and over 1000 gait trials. Subjects are numbered according to data source: self-collected subjects are designated M01 and M02, while literature data subjects are designated P01, P02, and P03.

The self-collection experiment recruited two healthy adult subjects. M01 was a 25-year-old female, 175 cm in height and 62.65 kg in body weight; M02 was a 28-year-old male, 172 cm in height and 70 kg in body weight. Neither subject had a history of lower-limb motor dysfunction, and both signed informed consent forms prior to the experiment. For M01, three walking speed conditions were established—slow (0.4 m/s), normal (0.8 m/s), and fast (1.2 m/s)—with 100 complete gait cycles collected under each condition. M02 completed 20 gait cycles at a single speed of 0.7 m/s as supplementary validation. A metronome was used throughout to assist with pace control.

The public gait dataset published by Rosenberg et al. [22] was additionally incorporated, with data from three subjects (P01, P02, P03) selected, covering a high-speed range of 1.30–1.45 m/s and comprising 230 trials. A summary of subjects and experimental conditions is provided in Table 3.

The subject-specific segment parameters used in simulation are listed in Table 4 and Table 5. Human segment masses were estimated from Winter’s anthropometric data [23], scaled proportionally to each subject’s body weight. Segment lengths were obtained from direct anthropometric measurements for self-collected subjects and estimated from the reported height using standard anthropometric ratios [23] for public-dataset subjects. The segment masses of the thigh, shank, and foot represent single-limb values. Exoskeleton component masses were obtained by weighing and rounded to the nearest 0.5 kg and are identical across subjects. System mass represents the sum of the human segment mass and the corresponding exoskeleton component mass.

#### 3.1.3. Data Preprocessing

The raw 3D marker trajectory data recorded by the Vicon system were first processed in Vicon Nexus 2.16 (Vicon Motion Systems Ltd., Oxford, UK) for marker labelling, gap filling, and trajectory reconstruction. The corresponding joint angle data were then exported for subsequent analysis in MATLAB R2022a (MathWorks, Inc., Natick, MA, USA). The exported joint angle trajectories were filtered using a fourth-order Butterworth low-pass filter with a cutoff frequency of 8 Hz to reduce high-frequency noise. Gait cycles were identified based on right-foot heel-strike events, and each cycle was time-normalised to 0–100% of the gait cycle. The ensemble-averaged joint angle profiles across all valid cycles were used as the desired trajectory input q_d_(t) for subsequent control simulations.

**Figure 4:**
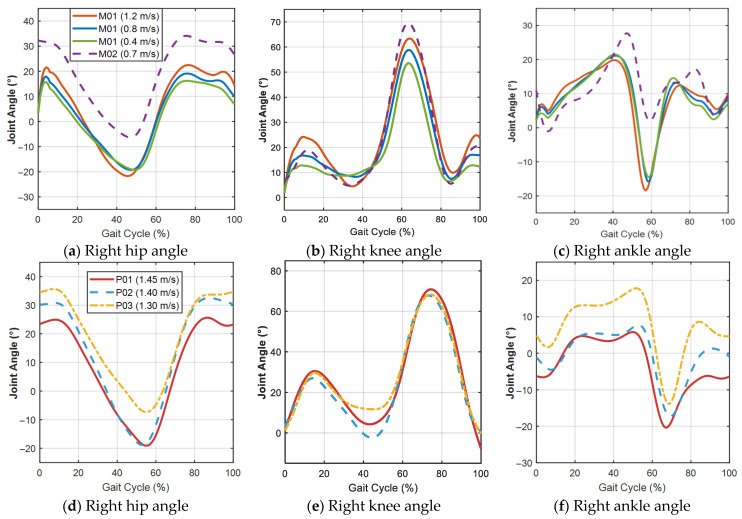
Mean joint angle trajectories during the gait cycle.

The resulting joint angle profiles are presented in Figure 4. Due to bilateral symmetry, only the right lower-limb data are shown. Each curve represents the ensemble average over all valid gait cycles for the corresponding subject and walking condition. Figure 4a–c presents the right hip, knee, and ankle joint angle trajectories of the self-collected subjects M01 (at 0.4, 0.8, and 1.2 m/s) and M02 (at 0.7 m/s). Figure 4d–f presents the corresponding trajectories of the public-dataset subjects P01, P02, and P03. For P01-P03, the joint angle data were obtained from the publicly available dataset reported by Rosenberg et al. [22]. Since the public dataset directly provides joint angle trajectories, these data were processed using the same subsequent procedure as the self-collected data, including filtering, gait cycle segmentation, normalisation, and ensemble averaging.

### 3.2. Control Strategy Comparison

#### 3.2.1. Simulation Setup and Evaluation Metrics

Simulations were conducted in the MATLAB R2022a environment with a simulation step size of 0.01 s. A typical gait cycle at the corresponding walking speed was selected as the desired trajectory for each subject. Random perturbations of ±5° were applied to the initial system state on the basis of the desired joint angles to examine the tracking performance of different control strategies.

Control performance was evaluated using the following metrics:

(1) Root Mean Square Error (RMSE): An overall measure of tracking error, calculated as $\mathrm{RMSE}=\sqrt{\frac{1}{N}\sum_{i=1}^{N}e_{i}^{2}}$;
(2) Maximum Error (ME): The maximum instantaneous deviation during tracking, calculated as $e_{\max}=\max|e_{i}|$;
(3) Steady-State Error (SSE): The mean absolute error during the final 10% of the gait cycle, representing the system’s steady-state tracking accuracy;
(4) Control Torque RMS: An indicator of the controller’s energy consumption level.

#### 3.2.2. Overall Tracking Performance Comparison

**Figure 5:**
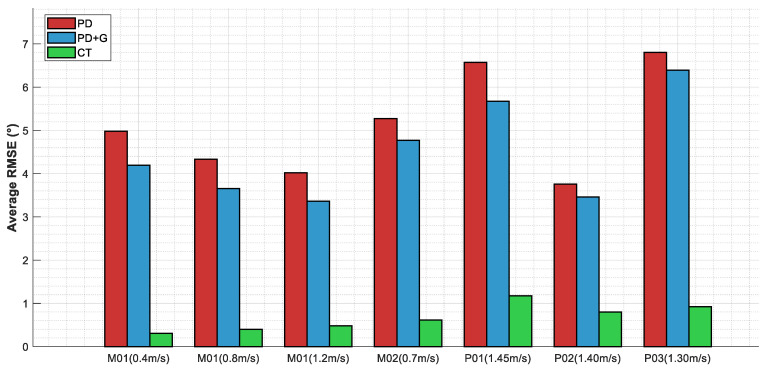
Comparison of tracking accuracy across different control strategies.

**Figure 6:**
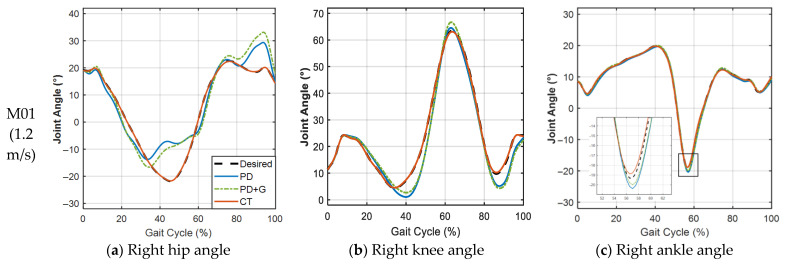

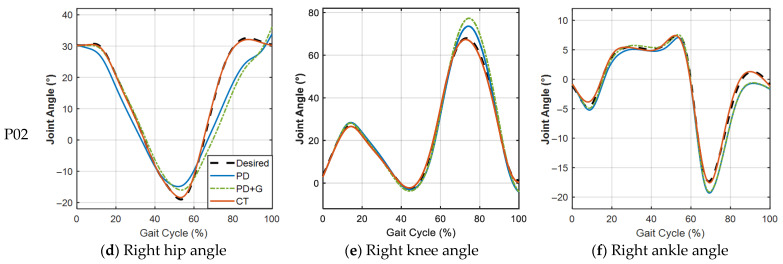
Joint-angle tracking results under three control strategies for subjects M01 and P02.

In the following figures and tables, PD denotes Proportional–Derivative control, PD + G denotes PD control with gravity compensation, and CT denotes the computed torque method, as detailed in Section 2.3.1, Section 2.3.2 and Section 2.3.3. Figure 5 presents the mean RMSE comparison across three control strategies for all seven subjects under different walking speed conditions. To provide an intuitive illustration of the trajectory tracking performance, Figure 6 presents representative joint angle tracking results under the three control strategies. Table 6 summarises the corresponding quantitative statistical results. The improvement ratio denotes the percentage reduction in RMSE relative to PD control.

Figure 6a–c shows the right hip, knee, and ankle angle tracking for subject M01 at 1.2 m/s (ensemble average of 100 cycles), and Figure 6d–f shows the corresponding results for subject P02 at 1.40 m/s (ensemble average of 230 trials). Under PD control, noticeable deviations from the desired trajectory are observed, particularly at the hip and knee joints. The computed torque method (CT) achieves close agreement with the desired trajectory across all joints.

Within the speed range of 0.4–1.45 m/s, PD control yielded mean RMSE values of 3.76–6.80°, with maximum errors as high as 8.94–16.38° and steady-state errors of 2.47–5.66°. PD with gravity compensation achieved RMSE values of 3.46–6.39°, representing improvements of 6.0–16.4% over PD control, with moderate performance gains under certain conditions. The computed torque method achieved RMSE values of 0.31–1.17°, maximum errors of 0.67–3.70°, and steady-state errors of 0.27–0.53°, with improvement ratios of 78.7–93.8% over PD control. The mean improvement ratio was 86.9%, with a standard deviation of only 4.8% across the seven conditions, indicating consistently stable performance. Regarding control torque, the RMS values for PD control ranged from 24.72 to 46.30 N·m, while those for the computed torque method ranged from 4.16 to 23.44 N·m, representing a reduction in energy consumption of 44–84%.

#### 3.2.3. Joint-Level Performance Analysis

**Figure 7:**
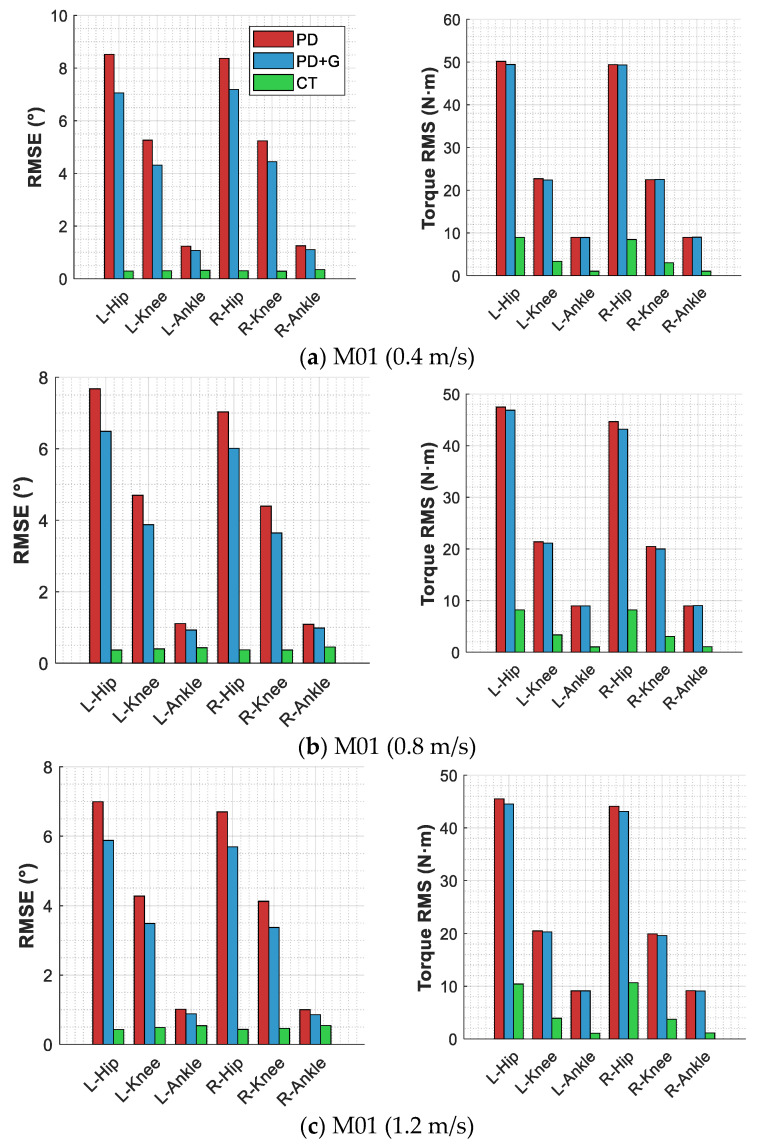

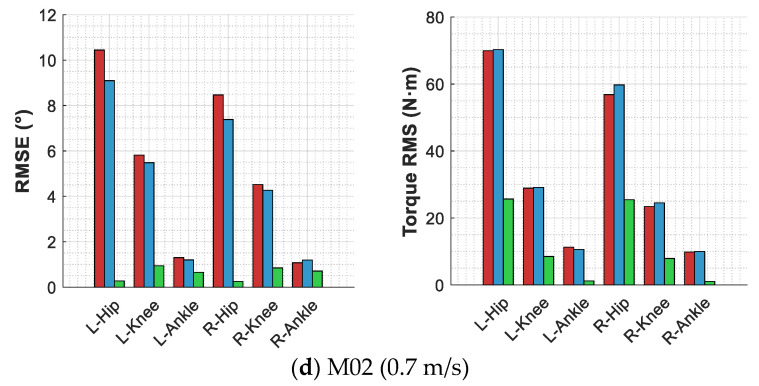
Per-joint tracking accuracy and control torque of three control strategies for self-collected subjects.

**Figure 8:**
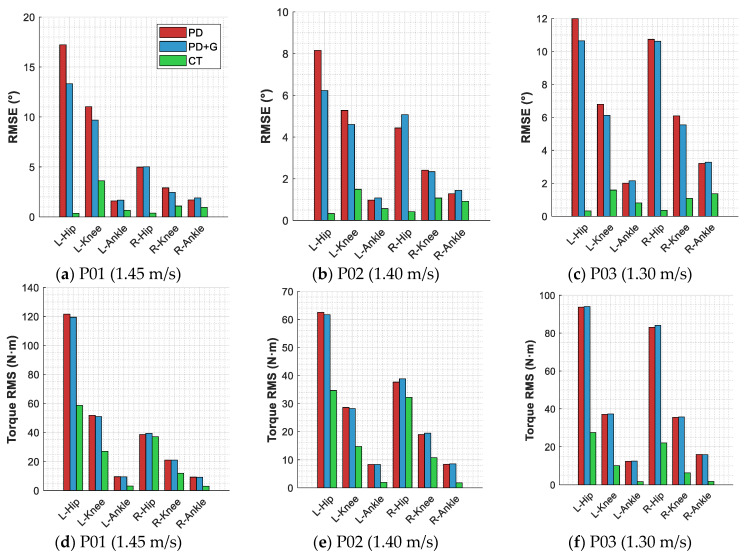
Per-joint tracking accuracy and control torque of three control strategies for literature data subjects.

Figure 7 and Figure 8 present the per-joint RMSE and control torque RMS distributions, with self-collected subjects (M01 and M02) shown in Figure 7 and literature data subjects (P01-P03) in Figure 8. Table 7 lists the per-joint RMSE statistics under representative speed conditions for each subject.

The per-joint data across all subjects reveal a consistent pattern: under PD control, the hip joint exhibits the largest errors—with left-hip RMSE values ranging from 7.68° to 17.23° across subjects—followed by the knee joint, while the ankle joint shows the smallest errors. The computed torque method yields a more uniform error distribution across joints; for self-collected subjects M01 and M02, the RMSE values at all six joints remain below 1°. Among the literature data subjects, P01 exhibits a notably elevated left-knee error of 3.62°, which is substantially higher than that of other joints, and the left-side hip and knee errors (0.36° and 3.62°) are significantly larger than their right-side counterparts (0.38° and 1.08°), exhibiting a left-right asymmetry. P02 and P03 also display left-right asymmetry to varying degrees. This is attributable to the experimental setup of the dataset, in which subjects walked while wearing bilateral passive ankle exoskeletons under the K0 (zero-torque) condition. Although no active torque was applied, the added mass and structural constraints of the exoskeleton affected ankle joint kinematics, which propagated through the kinematic chain to influence hip and knee dynamics, introducing differences from free walking. The left-right asymmetry may be related to individual compensation strategies. Regarding control torque, under PD control the hip joint requires the highest torque demand—with the left-hip torque RMS for P01 reaching as high as 121.58 N·m—whereas the computed torque method substantially reduces the torque at all joints, with ankle joint torque reduced to 1–3 N·m across all subjects and hip joint torque reduced by 44–88%.

#### 3.2.4. Statistical Distribution Analysis

**Figure 9:**
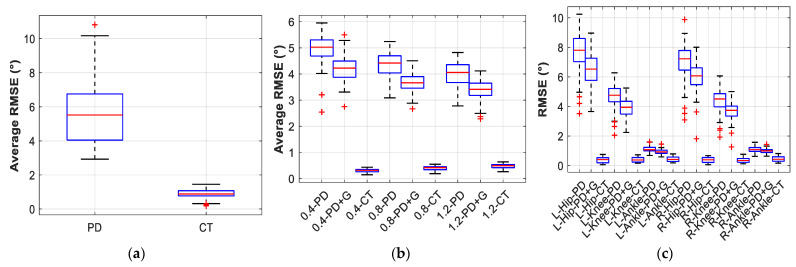

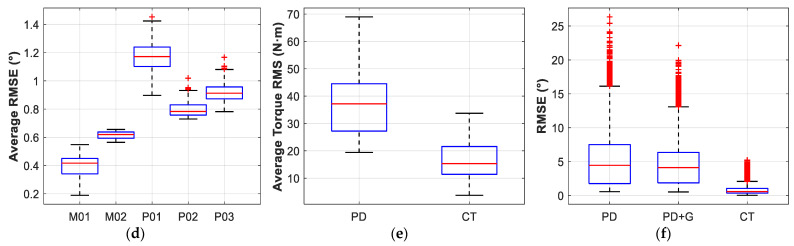
Statistical performance analysis of three control strategies from multiple perspectives. (**a**) Overall RMSE distribution under PD control and the computed torque method across all subjects; (**b**) RMSE distribution of three control strategies at three walking speeds for subject M01; (**c**) Per-joint RMSE distribution of three control strategies for subject M01 at 0.8 m/s; (**d**) Inter-subject RMSE variability under the computed torque method; (**e**) Overall torque RMS distribution under PD control and the computed torque method across all subjects; (**f**) Overall RMSE distribution of three control strategies across all pooled data.

Figure 9 provides a comprehensive, multi-dimensional statistical analysis of the performance distributions of the three control methods. The ‘+’ markers denote outliers, defined as data points exceeding 1.5 times the interquartile range beyond the upper or lower quartile.

Figure 9a summarises the overall RMSE distributions under the computed torque method and PD control across all subjects; the median RMSE for the computed torque method is approximately 0.40°, with an interquartile range of approximately 0.30°, whereas the median for PD control is 4.33°, with an interquartile range exceeding 2° and multiple outliers above 8°. Figure 9b shows the distributions for three speed conditions of subject M01 under the three methods, with the three CT boxes all below 0.5° and compact in form. Figure 9c presents a fine-grained distribution of 18 combinations (6 joints × 3 methods) for subject M01 at 0.8 m/s; all 18 CT boxes remain below 0.5° with good consistency across joints, while PD control exhibits pronounced inter-joint stratification with hip-joint boxes at a median of approximately 7.5° and ankle-joint boxes at approximately 1.1°. Figure 9d compares inter-individual variability under the computed torque method: M01’s boxes across speed conditions are concentrated between 0.3° and 0.5°, while those for P01-P03 are concentrated between 0.8° and 1.2°, with a clear difference between the two groups. Figure 9e shows the overall torque RMS distributions; the median torque for CT is substantially lower than that for PD control. Figure 9f encompasses more than 6000 sample points; the medians for PD, PD + G, and CT are 4.33°, 4.16°, and 0.40°, respectively, and the upper quartile of the CT distribution (0.60°) falls below the lower quartile of the PD distribution (3.50°), such that the two distributions are almost completely separated.

#### 3.2.5. Accuracy–Energy Consumption Trade-Off Analysis

**Figure 10:**
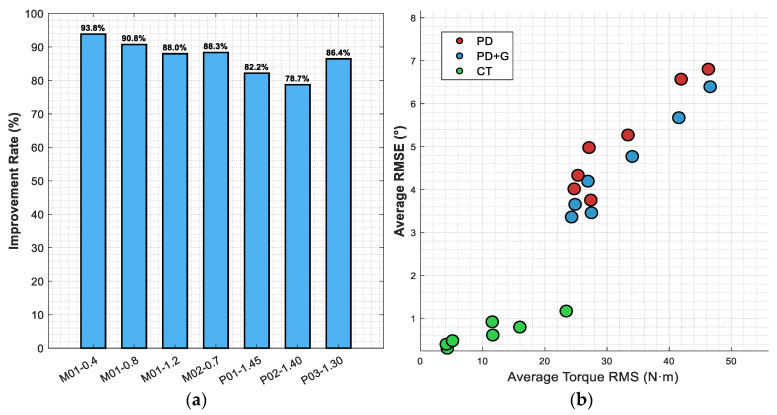
Improvement rate of the computed torque method and accuracy–energy consumption trade-off analysis. (**a**) Improvement rate of the computed torque method over PD control; (**b**) Accuracy–energy consumption trade-off of three control strategies across all subjects.

Figure 10a presents the distribution of improvement ratios of the computed torque method over PD control under each subject condition; Figure 10b displays the accuracy–energy consumption trade-off relationship across all subjects and three control strategies in the form of a scatter plot. In subfigure (b), the red, blue, and green data points represent PD control, PD + G, and the computed torque method (CT), respectively.

In Figure 10a, the improvement ratios for subject M01 at the three speed conditions are 93.8%, 90.8%, and 88.0%, showing a slight decreasing trend with increasing walking speed. Improvement ratios for the literature data subjects range from 78.7% to 86.4%, with a standard deviation of 4.8% across seven conditions, indicating that the computed torque method maintains stable performance improvements across different individuals and walking speeds. In Figure 10b, PD control data points are concentrated in the upper-right region (torque 20–50 N·m, RMSE 3–7°), while computed torque method points are concentrated in the lower-left region (torque 4–24 N·m, RMSE 0.3–1.2°), with the two clusters almost non-overlapping. PD + G data points are highly coincident with the PD cluster, with a clear gap between them and the CT cluster. The connecting lines across the three methods for each subject all display a pronounced migration from upper-right to lower-left, demonstrating that the performance improvements from PD to the computed torque method are achieved simultaneously in both accuracy and energy consumption dimensions.

**Figure 11:**
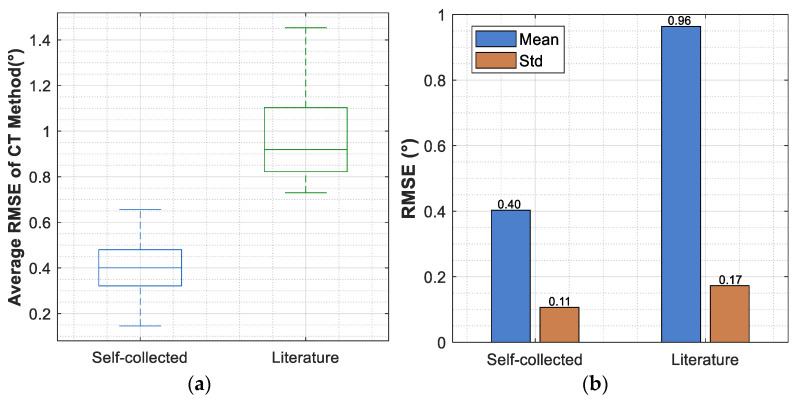
Tracking accuracy of the computed torque method across different data sources. (**a**) RMSE distribution of the computed torque method by data source; (**b**) Mean and standard deviation of tracking RMSE by data source.

Figure 11 compares the performance difference between self-collected data and literature data under the computed torque method; the mean RMSE for self-collected data is 0.45 ± 0.12°, while that for literature data is 0.96 ± 0.18°.

## 4. Discussion

This paper addresses the precise trajectory tracking control of lower-limb exoskeleton systems by establishing a 9-DOF dynamic model in the sagittal plane, designing three progressively structured control strategies, and conducting systematic simulation validation based on both self-collected and publicly available gait data. This section provides an in-depth discussion of the results from three perspectives: model validity, control performance, and research limitations, along with comparisons with existing studies.

### 4.1. Validity of the Dynamic Model

The 9-DOF model established in this paper offers a more complete kinematic description than the low-DOF models found in the existing literature. The 2-DOF models employed by Chen et al. [4] and Wu et al. [5] account only for the hip and knee joints, while the 4-DOF model of Yan et al. [7] similarly excludes the ankle joint and pelvic translational degrees of freedom. The present model simultaneously incorporates bilateral hip, knee, and ankle joint angles as well as horizontal and vertical pelvic translation within the sagittal plane, enabling a more complete description of the dynamic coupling between centre-of-mass displacement and joint motion during gait.

The mean RMSE of the computed torque method is 0.45 ± 0.12° on self-collected data (M01, M02) and 0.96 ± 0.18° on literature data (P01–P03), both representing high tracking accuracy—indirectly validating the accuracy of the dynamic model in characterising system inertia, Coriolis forces, and gravitational properties. Notably, the three control methods form a progressive relationship in their utilisation of the dynamic model: PD control, which is entirely model-independent, yields RMSE values as high as 3.76–6.80°; the introduction of gravity compensation produces limited improvement (PD + G RMSE: 3.46–6.39°); while the computed torque method, which fully exploits the complete dynamic model, reduces RMSE to 0.31–1.17°, with improvement ratios of 78.7–93.8%. This progressive improvement in performance substantiates that model accuracy is the core driver of control performance enhancement, further validating the reasonableness and practical value of the 9-DOF modelling approach.

### 4.2. Control Strategy Performance Analysis

Accuracy advantage of the computed torque method. By linearising the nonlinear dynamic system, the computed torque method achieves asymptotically stable trajectory tracking. It yields a mean RMSE of 0.59°, representing an 86.3% improvement over PD control (4.33°). This aligns with the accuracy improvements reported by Yu et al. [17] for active exoskeletons. Per-joint analysis reveals that PD control exhibits the largest errors at the hip joint (left-hip RMSE: 7.68–17.23°). This is expected, as the hip bears the largest inertial load and experiences the strongest time-varying gravitational moment. By accurately compensating for inertial and Coriolis forces, the computed torque method ensures a more uniform distribution of errors across all joints. Notably, RMSE values for all six joints in the self-collected subjects remained below 1°. This demonstrates how nonlinear compensation significantly mitigates the effects of the hip joint’s large inertia.

Limitations of PD with gravity compensation. While PD with gravity compensation improves performance by 6.0–16.4% over standard PD control, this enhancement is substantially lower than that of the computed torque method. This indicates that compensating solely for gravity while neglecting inertial and Coriolis forces limits performance during high-speed or complex dynamic motions. Consequently, as long as model parameter accuracy is ensured, a complete nonlinear compensation scheme provides significantly greater performance gains.

Co-optimisation of accuracy and energy consumption. While substantially enhancing tracking accuracy, the computed torque method also lowers the control torque RMS to 4.16–23.44 N·m. Compared to standard PD control (24.72–46.30 N·m), this represents a substantial energy consumption reduction of 44–84%. These findings align with Tucker et al. [20], confirming that precise dynamic modelling inherently reduces control energy demands. Ultimately, this synergy of high precision and energy efficiency provides crucial technical support for the endurance optimisation and lightweight design of exoskeleton systems.

Speed, adaptability, and generalisation capability. Across a walking speed range of 0.4–1.45 m/s and seven experimental conditions, the computed torque method maintains stable tracking without requiring control parameter adjustments. The standard deviation of its improvement ratios is only 4.8%, indicating excellent speed adaptability. Although the joint range of motion increases substantially with walking speed, the computed torque method effectively compensates for these dynamic changes. This further validates the generalisation capability of both the proposed model and the control strategy.

### 4.3. Performance Differences Between Self-Collected Subjects and Public-Dataset Subjects

The tracking accuracy of the self-collected subjects (M01, M02) under the computed torque method (0.45 ± 0.12°) is superior to that of the literature data subjects (0.96 ± 0.18°). The differences primarily arise from two sources. First, the literature dataset (Rosenberg et al. [22]) was collected from subjects walking while wearing bilateral passive ankle exoskeletons under the K0 condition; the added mass and structural constraints of the exoskeletons altered ankle joint kinematics and influenced hip and knee dynamics through the kinematic chain. Since the dynamic model parameters in this paper were set based on free-walking conditions, discrepancies exist between the model and the actual dynamics. Second, the literature subjects walked at relatively high speeds (1.30–1.45 m/s), where nonlinear dynamic effects are more pronounced and model accuracy requirements are correspondingly higher. The left-right asymmetry observed for P01—with left-side hip and knee errors (0.36° and 3.62°) being notably larger than the right-side values (0.38° and 1.08°)—may be related to asymmetric individual compensation strategies adopted when wearing the exoskeleton. These findings suggest that, in practical exoskeleton control applications, online model parameter identification or adaptive correction for the wearing state would be necessary to further enhance control accuracy across individuals and conditions.

### 4.4. Limitations and Future Work

The present study has the following limitations, which are to be addressed in future work:

(1) Simplifying model assumptions. This paper constrains the trunk to a vertical posture (θ_1_ = 90°) and simplifies the human–machine interaction to unidirectional ideal assistance, without accounting for human–machine contact forces, friction, or limb flexibility in actual wearing conditions. In particular, innate differences in joint flexibility across individuals are only implicitly captured through the subject-specific desired trajectories derived from motion capture data, rather than being explicitly modelled. Future work may incorporate flexible body modelling, passive joint impedance characterisation, or contact force models to improve system description accuracy.
(2) Limited number of subjects. The self-collection experiment involved only two healthy subjects, which is insufficient to adequately represent individuals of diverse body types, ages, and gait characteristics. Subsequent research should expand the subject sample, particularly by including the target rehabilitation population (e.g., elderly individuals, patients with gait disorders) for validation.
(3) Gap between simulation and physical verification. The conclusions of this paper are based on MATLAB simulation validation and have not yet been experimentally verified on a physical exoskeleton platform. Practical factors such as sensor noise, actuator delay, and mechanical elasticity may affect control performance; subsequent work should further evaluate the proposed methods on a physical platform.
(4) Extension of control strategies. The computed torque method is highly dependent on model accuracy and has limited robustness under parameter uncertainties or external disturbances. Future work may build upon this foundation to incorporate adaptive control, sliding mode control, or neural network compensation so as to further enhance robustness in practical applications. The neural network-based robust computed torque control framework proposed by Han et al. [19] offers a useful reference in this regard.

## 5. Conclusions

This paper addresses dynamic modelling and high-precision trajectory tracking control for lower-limb exoskeleton systems. A human–machine coupled dynamic model encompassing nine generalised coordinates in the sagittal plane was established, three progressively structured control strategies were designed, and systematic simulation validation was conducted based on both self-collected and publicly available gait data. The main conclusions are as follows:

(1) Based on the modified D-H parameter method and Lagrange’s equations, a lower-limb exoskeleton dynamic model with nine degrees of freedom—comprising bilateral hip, knee, and ankle joint angles together with horizontal and vertical pelvic translation—was established. Compared with traditional 2–4 DOF models, this model more comprehensively describes the coupling relationship between joint motion and centre-of-mass translation during gait dynamics, providing a more accurate theoretical foundation for control strategy design.
(2) The comparative results of the three control strategies demonstrate a positive correlation between the degree of dynamic model utilisation and control performance. By fully exploiting the mass matrix, Coriolis force term, and gravity term for nonlinear compensation, the computed torque method achieves a joint angle tracking RMSE of 0.59°, representing an 86.3% improvement over PD control, with a control torque RMS of only 4.44 N·m, simultaneously achieving co-optimisation in both tracking accuracy and energy consumption.
(3) Within the broad walking speed range of 0.4–1.45 m/s, the computed torque method maintains stable tracking performance without adjustment of control parameters, with a standard deviation of improvement ratios of only 4.8% across seven experimental conditions, validating the strong adaptability of the proposed model and control strategies to variations in walking speed and inter-individual differences.

In summary, high-precision dynamic modelling is the key to achieving high-performance trajectory tracking control in lower-limb exoskeleton systems. The 9-DOF modelling scheme and computed torque control strategy proposed in this paper can provide a theoretical basis and design reference for controller design, drive system selection, and endurance optimisation of lower-limb exoskeleton systems, and thus hold practical engineering value.

## Figures and Tables

**Table 1:** D-H parameters for human body segments.

i	α_i − 1_	a_i − 1_	d_i_	θ_i_	Joint/Segment
1	0	0	0	θ_body_	Trunk
2	0	0	0	θ_l_hip_	Left hip
3	0	l_l_thigh_	0	θ_l_knee_	Left knee
4	0	l_l_shank_	0	θ_l_ankle_	Left ankle
5	0	0	0	θ_r_hip_	Right hip
6	0	l_r_thigh_	0	θ_r_knee_	Right knee
7	0	l_r_shank_	0	θ_r_ankle_	Right ankle

**Table 2:** Controller gain parameters.

Joint	K_p_ (N·m/rad)	K_d_ (N·m·s/rad)
Hip joint	400	40
Knee joint	300	30
Ankle joint	200	20

**Table 3:** Summary of subjects and experimental conditions.

Subject	Data Source	Walking Speed (m/s)	No. of Trials	Notes
M01	Self-collected	0.4/0.8/1.2	100/100/100	Multi-speed complete dataset
M02	Self-collected	0.7	20	Single-speed supplementary validation
P01	Publicly available dataset	1.45	230	Rosenberg et al. [22]
P02	Publicly available dataset	1.40	230	Rosenberg et al. [22]
P03	Publicly available dataset	1.30	230	Rosenberg et al. [22]

**Table 4:** Subject-specific segment parameters used in simulation for self-collected subjects.

Segment	Exo. Mass (kg)	M01 (62.65 kg, 1.75 m)		M02 (70.0 kg, 1.72 m)	
		Human Mass (kg)	Length (m)	Human Mass (kg)	Length (m)
Trunk	—	30.45	—	34.02	—
Thigh	1.50	6.27	0.456	7.00	0.448
Shank	1.20	2.91	0.403	3.26	0.396
Foot	0.50	0.91	0.245	1.02	0.255

**Table 5:** Subject-specific segment parameters used in simulation for public-dataset subjects.

Segment	Exo. Mass (kg)	P01 (73.5 kg, 1.778 m)		P02 (61.24 kg, 1.651 m)		P03 (49.9 kg, 1.60 m)	
		Human Mass (kg)	Length (m)	Human Mass (kg)	Length (m)	Human Mass (kg)	Length (m)
Trunk	—	35.72	—	29.76	—	24.25	—
Thigh	1.50	10.41	0.462	9.05	0.439	4.99	0.446
Shank	1.20	3.18	0.463	2.95	0.441	2.32	0.394
Foot	0.50	1.01	0.270	0.79	0.251	0.72	0.245

**Table 6:** Comprehensive control performance statistics for each subject.

Subject (Speed)	Method	RMSE (°)	Max Error (°)	SSE (°)	Torque RMS (N·m)	Improvement
M01 (0.4 m/s)	PD	4.98 ± 3.08	11.57	5.00	27.10	---
	PD + G	4.20 ± 2.58	9.78	4.19	26.93	15.7%
	CT	0.31 ± 0.15	0.67	0.32	4.31	93.8%
M01 (0.8 m/s)	PD	4.33 ± 2.70	10.24	4.16	25.33	---
	PD + G	3.66 ± 2.28	8.96	3.71	24.86	15.5%
	CT	0.40 ± 0.18	0.81	0.43	4.16	90.8%
M01 (1.2 m/s)	PD	4.02 ± 2.51	8.95	3.71	24.72	---
	PD + G	3.36 ± 2.11	7.78	3.11	24.30	16.4%
	CT	0.48 ± 0.20	1.00	0.53	5.16	88.0%
M02 (0.7 m/s)	PD	5.27 ± 3.56	11.13	5.44	33.37	---
	PD + G	4.77 ± 3.06	9.59	4.94	34.05	9.5%
	CT	0.61 ± 0.27	2.66	0.27	11.63	88.3%
P01 (1.45 m/s)	PD	6.57 ± 5.93	15.17	3.70	41.91	---
	PD + G	5.67 ± 4.57	13.01	3.53	41.54	13.7%
	CT	1.17 ± 1.15	3.70	0.42	23.44	82.2%
P02 (1.40 m/s)	PD	3.76 ± 2.61	8.94	2.47	27.39	---
	PD + G	3.46 ± 2.04	7.69	2.64	27.49	8.0%
	CT	0.80 ± 0.43	2.68	0.35	15.98	78.7%
P03 (1.30 m/s)	PD	6.80 ± 4.18	16.38	5.66	46.30	---
	PD + G	6.39 ± 3.86	15.22	5.67	46.58	6.0%
	CT	0.92 ± 0.51	2.84	0.45	11.54	86.4%

**Table 7:** Per-joint RMSE comparison across subjects (unit: degree (°)).

Subject (Speed)	Method	L. Hip	L. Knee	L. Ankle	R. Hip	R. Knee	R. Ankle	Mean
M01 (0.8 m/s)	PD	7.68	4.70	1.11	7.03	4.39	1.09	4.33
	PD + G	6.49	3.88	0.93	6.01	3.64	0.98	3.66
	CT	0.37	0.40	0.44	0.38	0.37	0.45	0.40
M02 (0.7 m/s)	PD	10.45	5.82	1.30	8.46	4.52	1.08	5.27
	PD + G	9.10	5.48	1.20	7.39	4.27	1.19	4.77
	CT	0.27	0.95	0.66	0.25	0.85	0.71	0.61
P01 (1.45 m/s)	PD	17.23	11.03	1.59	4.98	2.90	1.70	6.57
	PD + G	13.33	9.68	1.68	5.01	2.45	1.90	5.67
	CT	0.36	3.62	0.65	0.38	1.08	0.95	1.17
P02 (1.40 m/s)	PD	8.16	5.28	0.98	4.43	2.41	1.28	3.76
	PD + G	6.23	4.60	1.08	5.07	2.34	1.44	3.46
	CT	0.33	1.50	0.57	0.42	1.07	0.92	0.80
P03 (1.30 m/s)	PD	11.99	6.79	2.01	10.74	6.09	3.20	6.80
	PD + G	10.64	6.12	2.15	10.62	5.55	3.28	6.39
	CT	0.32	1.59	0.81	0.36	1.09	1.36	0.92

## Data Availability

Part of the data used in this study is publicly available from Rosenberg et al. [22]. Due to data privacy considerations, the self-collected dataset is not publicly available but may be obtained from the first author upon reasonable request. The code for the dynamic equations will be made publicly available in the future.

## Supplementary Materials

The following supporting information can be downloaded at: https://www.mdpi.com/article/10.3390/s26072124/s1, Code S1: lowerLimbKinematics.m: MATLAB script for the kinematic model of the lower-limb exoskeleton; Code S2: lowerLimbDynamics.m: MATLAB script for deriving the dynamic equations; Code S3: run_analysis.m: MATLAB script for executing the simulation.

## Appendix A

This appendix provides the complete symbolic expressions for the mass matrix M, Coriolis/centrifugal force vector C, and gravity vector G derived in Section 2.2. Due to the high dimensionality of the 9-DOF model, these expressions are omitted from the main text for readability. To improve compactness, composite trigonometric functions are abbreviated using the sum-to-product convention: $s_{ij}=\sin(\theta_{i}+\theta_{j})$, $c_{ij}=\cos(\theta_{i}+\theta_{j})$, and by extension $s_{234}=\sin(\theta_{2}+\theta_{3}+\theta_{4})$, $c_{234}=\cos(\theta_{2}+\theta_{3}+\theta_{4})$, with analogous definitions for all other composite angle terms. Single-angle terms follow $s_{i}=\sin\theta_{i}$, $c_{i}=\cos\theta_{i}$.

$$\begin{array}{l} M_{11}=\frac{{L_{\mathrm{body}}}^{2}m_{\mathrm{body}}}{3}\\ M_{12}=M_{21}=M_{13}=M_{31}=M_{14}=M_{41}=M_{15}=M_{51}=M_{16}=M_{61}=M_{17}=M_{71}=0\\ M_{18}=M_{81}-\frac{L_{body}m_{body}s_{1}}{2}\\ M_{19}=M_{91}=\frac{L_{body}m_{body}c_{1}}{2}\\ M_{22}=\frac{{L_{F}}^{2}m_{LF}}{3}+{L_{S}}^{2}m_{LF}+\frac{{L_{S}}^{2}m_{LS}}{3}+{L_{T}}^{2}m_{LF}+{L_{T}}^{2}m_{LS}+\frac{{L_{T}}^{2}m_{LT}}{3}+L_{F}L_{S}c_{4}m_{LF}+L_{F}L_{T}c_{34}m_{LF}+\\ \;2L_{S}L_{T}c_{3}m_{LF}+L_{S}L_{T}c_{3}m_{LS}\\ M_{23}=M_{32}=\frac{{L_{F}}^{2}m_{LF}}{3}+{L_{S}}^{2}m_{LF}+\frac{{L_{S}}^{2}m_{LS}}{3}+L_{F}L_{S}c_{4}m_{LF}+\frac{L_{F}L_{T}c_{34}m_{LF}}{2}+L_{S}L_{T}c_{3}m_{LF}+\frac{L_{S}L_{T}c_{3}m_{LS}}{2}\\ M_{24}=M_{42}=\frac{L_{F}m_{LF}\left(2L_{F}+3L_{S}c_{4}+3L_{T}c_{34}\right)}{6}\\ M_{45}=M_{53}=M_{36}=M_{63}=M_{37}=M_{73}=0\\ M_{28}=M_{82}=-\frac{L_{F}m_{LF}s_{234}}{2}-L_{S}m_{LF}s_{23}-\frac{L_{S}m_{LS}s_{23}}{2}-L_{T}m_{LF}s_{2}-L_{T}m_{LS}s_{2}-\frac{L_{T}m_{LT}s_{2}}{2}\\ M_{29}=M_{92}=\frac{L_{F}c_{234}m_{LF}}{2}+L_{S}c_{23}m_{LF}+\frac{L_{S}c_{23}m_{LS}}{2}+L_{T}c_{2}m_{LF}+L_{T}c_{2}m_{LS}+\frac{L_{T}c_{2}m_{LT}}{2}\\ M_{33}=\frac{{L_{F}}^{2}m_{LF}}{3}+{L_{S}}^{2}m_{LF}+\frac{{L_{S}}^{2}m_{LS}}{3}+L_{F}L_{S}c_{4}m_{LF}\\ M_{34}=M_{43}=\frac{L_{F}m_{LF}\left(2L_{F}+3L_{S}c_{4}\right)}{6}\\ M_{35}=M_{53}=M_{36}=M_{63}=M_{37}=M_{73}=0\\ M_{38}=M_{83}=-m_{LF}\left(\frac{L_{F}s_{234}}{2}+L_{S}s_{23}\right)-\frac{L_{S}m_{LS}s_{23}}{2}\\ M_{39}=M_{93}=m_{LF}\left(\frac{L_{F}c_{234}}{2}+L_{S}c_{23}\right)+\frac{L_{S}c_{23}m_{LS}}{2}\\ M_{44}=\frac{{L_{F}}^{2}m_{LF}}{3}\\ M_{45}=M_{54}=M_{46}=M_{64}=M_{47}=M_{74}=0\\ M_{48}=M_{84}=-\frac{L_{F}m_{LF}s_{234}}{2}\\ M_{49}=M_{94}=\frac{L_{F}c_{234}m_{LF}}{2}\\ M_{55}=\frac{{R_{F}}^{2}m_{RF}}{3}+{R_{S}}^{2}m_{RF}+\frac{{R_{S}}^{2}m_{RS}}{3}+{R_{T}}^{2}m_{RF}+{R_{T}}^{2}m_{RS}+\frac{{R_{T}}^{2}m_{RT}}{3}+R_{F}R_{S}c_{7}m_{RF}+R_{F}R_{T}c_{67}m_{RF}+\\ \;2R_{S}R_{T}c_{6}m_{RF}+R_{S}R_{T}c_{6}m_{RS}\\ M_{56}=M_{65}=\frac{{R_{F}}^{2}m_{RF}}{3}+{R_{S}}^{2}m_{RF}+\frac{{R_{S}}^{2}m_{RS}}{3}+R_{F}R_{S}c_{7}m_{RF}+\frac{R_{F}R_{T}c_{67}m_{RF}}{2}+R_{S}R_{T}c_{6}m_{RF}+\frac{R_{S}R_{T}c_{6}m_{RS}}{2}\\ M_{57}=M_{75}=\frac{R_{F}m_{RF}\left(2R_{F}+3R_{S}c_{7}+3R_{T}c_{67}\right)}{6}\\ M_{58}=M_{85}=-\frac{R_{F}m_{RF}s_{567}}{2}-R_{S}m_{RF}s_{56}-\frac{R_{S}m_{RS}s_{56}}{2}-R_{T}m_{RF}s_{5}-R_{T}m_{RS}s_{5}-\frac{R_{T}m_{RT}s_{5}}{2}\\ M_{59}=M_{95}=\frac{R_{F}c_{567}m_{RF}}{2}+R_{S}c_{56}m_{RF}+\frac{R_{S}c_{56}m_{RS}}{2}+R_{T}c_{5}m_{RF}+R_{T}c_{5}m_{RS}+\frac{R_{T}c_{5}m_{RT}}{2}\\ M_{66}=\frac{{R_{F}}^{2}m_{RF}}{3}+{R_{S}}^{2}m_{RF}+\frac{{R_{S}}^{2}m_{RS}}{3}+R_{F}R_{S}c_{7}m_{RF}\\ M_{67}=M_{76}=\frac{R_{F}m_{RF}\left(2R_{F}+3R_{S}c_{7}\right)}{6}\\ M_{68}=M_{86}=-m_{RF}\left(\frac{R_{F}s_{567}}{2}+R_{S}s_{56}\right)-\frac{R_{S}m_{RS}s_{56}}{2}\\ M_{69}=M_{96}=m_{RF}\left(\frac{R_{F}c_{567}}{2}+R_{S}c_{56}\right)+\frac{R_{S}c_{56}m_{RS}}{2}\\ M_{77}=\frac{{R_{F}}^{2}m_{RF}}{3}\\ M_{78}=M_{87}=-\frac{R_{F}m_{RF}s_{567}}{2}\\ M_{79}=M_{97}=\frac{R_{F}c_{567}m_{RF}}{2}\\ M_{88}=m_{\mathrm{LF}}{+m}_{\mathrm{LS}}{+m}_{\mathrm{LT}}{+m}_{\mathrm{RF}}{+m}_{\mathrm{RS}}{+m}_{\mathrm{RT}}{+m}_{\mathrm{body}}\\ M_{89}={\;M}_{98}=0\\ M_{99}=m_{\mathrm{LF}}{+m}_{\mathrm{LS}}{+m}_{\mathrm{LT}}{+m}_{\mathrm{RF}}{+m}_{\mathrm{RS}}{+m}_{\mathrm{RT}}{+m}_{\mathrm{body}}\\ C_{1}=0\\ C_{2}=-\frac{L_{F}L_{S}{{\dot{\theta }}_{4}}^{2}m_{LF}s_{4}}{2}-\frac{L_{F}L_{T}{{\dot{\theta }}_{3}}^{2}m_{LF}s_{34}}{2}-\frac{L_{F}L_{T}{{\dot{\theta }}_{4}}^{2}m_{LF}s_{34}}{2}-L_{S}L_{T}{{\dot{\theta }}_{3}}^{2}m_{LF}s_{3}-\frac{L_{S}L_{T}{{\dot{\theta }}_{3}}^{2}m_{LS}s_{3}}{2}-\\ \;L_{F}L_{S}{\dot{\theta }}_{2}{\dot{\theta }}_{4}m_{LF}s_{4}-L_{F}L_{S}{\dot{\theta }}_{3}{\dot{\theta }}_{4}m_{LF}s_{4}-L_{F}L_{T}{\dot{\theta }}_{2}{\dot{\theta }}_{3}m_{LF}s_{34}-L_{F}L_{T}{\dot{\theta }}_{2}{\dot{\theta }}_{4}m_{LF}s_{34}-\\ \;L_{F}L_{T}{\dot{\theta }}_{3}{\dot{\theta }}_{4}m_{LF}s_{34}-2L_{S}L_{T}{\dot{\theta }}_{2}{\dot{\theta }}_{3}m_{LF}s_{3}-L_{S}L_{T}{\dot{\theta }}_{2}{\dot{\theta }}_{3}m_{LS}s_{3}\\ C_{3}=\frac{L_{F}L_{T}{{\dot{\theta }}_{2}}^{2}m_{LF}s_{34}}{2}-\frac{L_{F}L_{S}{{\dot{\theta }}_{4}}^{2}m_{LF}s_{4}}{2}+L_{S}L_{T}{{\dot{\theta }}_{2}}^{2}m_{LF}s_{3}+\frac{L_{S}L_{T}{{\dot{\theta }}_{2}}^{2}m_{LS}s_{3}}{2}-L_{F}L_{S}{\dot{\theta }}_{2}{\dot{\theta }}_{4}m_{LF}s_{4}-\\ \;L_{F}L_{S}{\dot{\theta }}_{3}{\dot{\theta }}_{4}m_{LF}s_{4}\\ C_{4}=\frac{L_{F}m_{LF}\left(L_{S}{{\dot{\theta }}_{2}}^{2}s_{4}+L_{S}{{\dot{\theta }}_{3}}^{2}s_{4}+L_{T}{{\dot{\theta }}_{2}}^{2}s_{34}+2L_{S}{\dot{\theta }}_{2}{\dot{\theta }}_{3}s_{4}\right)}{2}\\ C_{5}=-\frac{R_{F}R_{T}{{\dot{\theta }}_{7}}^{2}m_{RF}s_{7}}{2}-\frac{R_{F}R_{T}{{\dot{\theta }}_{6}}^{2}m_{RF}s_{67}}{2}-\frac{R_{F}R_{T}{{\dot{\theta }}_{7}}^{2}m_{RF}s_{67}}{2}-R_{S}R_{T}{{\dot{\theta }}_{6}}^{2}m_{RF}s_{6}-\frac{R_{S}R_{T}{{\dot{\theta }}_{6}}^{2}m_{RS}s_{6}}{2}\\ \;-R_{F}R_{S}{\dot{\theta }}_{5}{\dot{\theta }}_{7}m_{RF}s_{7}-R_{F}R_{S}{\dot{\theta }}_{6}{\dot{\theta }}_{7}m_{RF}s_{7}-R_{F}R_{T}{\dot{\theta }}_{5}{\dot{\theta }}_{6}m_{RF}s_{67}-R_{F}R_{T}{\dot{\theta }}_{5}{\dot{\theta }}_{7}m_{RF}s_{67}-\\ \;R_{F}R_{T}{\dot{\theta }}_{6}{\dot{\theta }}_{7}m_{RF}s_{67}-2R_{S}R_{T}{\dot{\theta }}_{5}{\dot{\theta }}_{6}m_{RF}s_{6}-R_{S}R_{T}{\dot{\theta }}_{5}{\dot{\theta }}_{6}m_{RS}s_{6}\\ C_{6}=\frac{R_{F}R_{T}{{\dot{\theta }}_{5}}^{2}m_{RF}s_{67}}{2}-\frac{R_{F}R_{S}{{\dot{\theta }}_{7}}^{2}m_{RF}s_{7}}{2}+R_{S}R_{T}{{\dot{\theta }}_{5}}^{2}m_{RF}s_{6}+\frac{R_{S}R_{T}{{\dot{\theta }}_{5}}^{2}m_{RS}s_{6}}{2}-R_{F}R_{S}{\dot{\theta }}_{5}{\dot{\theta }}_{7}m_{RF}s_{7}\\ \;-R_{F}R_{S}{\dot{\theta }}_{6}{\dot{\theta }}_{7}m_{RF}s_{7}\\ C_{7}=\frac{R_{F}m_{RF}\left(R_{S}{{\dot{\theta }}_{5}}^{2}s_{7}+R_{S}{{\dot{\theta }}_{6}}^{2}s_{7}+R_{T}{{\dot{\theta }}_{5}}^{2}s_{67}+2R_{S}{\dot{\theta }}_{5}{\dot{\theta }}_{6}s_{7}\right)}{2}\\ C_{8}=-\frac{L_{F}c_{234}{{\dot{\theta }}_{2}}^{2}m_{LF}}{2}-\frac{L_{F}c_{234}{{\dot{\theta }}_{3}}^{2}m_{LF}}{2}-\frac{L_{F}c_{234}{{\dot{\theta }}_{4}}^{2}m_{LF}}{2}-L_{S}c_{23}{{\dot{\theta }}_{2}}^{2}m_{LF}-L_{S}c_{23}{{\dot{\theta }}_{3}}^{2}m_{LF}-\frac{L_{S}c_{23}{{\dot{\theta }}_{2}}^{2}m_{LS}}{2}\\ \;-\frac{L_{S}c_{23}{{\dot{\theta }}_{3}}^{2}m_{LS}}{2}-L_{T}c_{2}{{\dot{\theta }}_{2}}^{2}m_{LF}-L_{T}c_{2}{{\dot{\theta }}_{2}}^{2}m_{LS}-\frac{L_{T}c_{2}{{\dot{\theta }}_{2}}^{2}m_{LT}}{2}-\frac{R_{F}c_{567}{{\dot{\theta }}_{5}}^{2}m_{RF}}{2}-\frac{R_{F}c_{567}{{\dot{\theta }}_{6}}^{2}m_{RF}}{2}\\ \;-\frac{R_{F}c_{567}{{\dot{\theta }}_{7}}^{2}m_{RF}}{2}-R_{S}c_{56}{{\dot{\theta }}_{5}}^{2}m_{RF}-R_{S}c_{56}{{\dot{\theta }}_{6}}^{2}m_{RF}-\frac{R_{S}c_{56}{{\dot{\theta }}_{5}}^{2}m_{RS}}{2}-\frac{R_{S}c_{56}{{\dot{\theta }}_{6}}^{2}m_{RS}}{2}-R_{T}c_{5}{{\dot{\theta }}_{5}}^{2}m_{RF}\\ \;-R_{T}c_{5}{{\dot{\theta }}_{5}}^{2}m_{RS}-\frac{R_{T}c_{5}{{\dot{\theta }}_{5}}^{2}m_{RT}}{2}-\frac{L_{body}{{\dot{\theta }}_{1}}^{2}m_{body}\cos\left(\theta_{1}\right)}{2}-L_{F}c_{234}{\dot{\theta }}_{2}{\dot{\theta }}_{3}m_{LF}-L_{F}c_{234}{\dot{\theta }}_{2}{\dot{\theta }}_{4}m_{LF}\\ \;-L_{F}c_{234}{\dot{\theta }}_{3}{\dot{\theta }}_{4}m_{LF}-2L_{S}c_{23}{\dot{\theta }}_{2}{\dot{\theta }}_{3}m_{LF}-L_{S}c_{23}{\dot{\theta }}_{2}{\dot{\theta }}_{3}m_{LS}-R_{F}c_{567}{\dot{\theta }}_{5}{\dot{\theta }}_{6}m_{RF}-R_{F}c_{567}{\dot{\theta }}_{5}{\dot{\theta }}_{7}m_{RF}\\ \;-R_{F}c_{567}{\dot{\theta }}_{6}{\dot{\theta }}_{7}m_{RF}-2R_{S}c_{56}{\dot{\theta }}_{5}{\dot{\theta }}_{6}m_{RF}-R_{S}c_{56}{\dot{\theta }}_{5}{\dot{\theta }}_{6}m_{RS}\\ C_{9}=-\frac{L_{F}{{\dot{\theta }}_{2}}^{2}m_{LF}s_{234}}{2}-\frac{L_{F}{{\dot{\theta }}_{3}}^{2}m_{LF}s_{234}}{2}-\frac{L_{F}{{\dot{\theta }}_{4}}^{2}m_{LF}s_{234}}{2}-L_{S}{{\dot{\theta }}_{2}}^{2}m_{LF}s_{23}-L_{S}{{\dot{\theta }}_{3}}^{2}m_{LF}s_{23}-\frac{L_{S}{{\dot{\theta }}_{2}}^{2}m_{LS}s_{23}}{2}\\ \;-\frac{L_{S}{{\dot{\theta }}_{3}}^{2}m_{LS}s_{23}}{2}-L_{T}{{\dot{\theta }}_{2}}^{2}m_{LF}s_{2}-L_{T}{{\dot{\theta }}_{2}}^{2}m_{LS}s_{2}-\frac{L_{T}{{\dot{\theta }}_{2}}^{2}m_{LT}s_{2}}{2}-\frac{R_{F}{{\dot{\theta }}_{5}}^{2}m_{RF}s_{567}}{2}-\frac{R_{F}{{\dot{\theta }}_{6}}^{2}m_{RF}s_{567}}{2}\\ \;-\frac{R_{F}{{\dot{\theta }}_{7}}^{2}m_{RF}s_{567}}{2}-R_{S}{{\dot{\theta }}_{5}}^{2}m_{RF}s_{56}-R_{S}{{\dot{\theta }}_{6}}^{2}m_{RF}s_{56}-\frac{R_{S}{{\dot{\theta }}_{5}}^{2}m_{RS}s_{56}}{2}-\frac{R_{S}{{\dot{\theta }}_{6}}^{2}m_{RS}s_{56}}{2}-R_{T}{{\dot{\theta }}_{5}}^{2}m_{RF}s_{5}\\ \;-R_{T}{{\dot{\theta }}_{5}}^{2}m_{RS}s_{5}-\frac{R_{T}{{\dot{\theta }}_{5}}^{2}m_{RT}s_{5}}{2}-\frac{L_{body}{{\dot{\theta }}_{1}}^{2}m_{body}\sin\left(\theta_{1}\right)}{2}-L_{F}{\dot{\theta }}_{2}{\dot{\theta }}_{3}m_{LF}s_{234}-L_{F}{\dot{\theta }}_{2}{\dot{\theta }}_{4}m_{LF}s_{234}\\ \;-L_{F}{\dot{\theta }}_{3}{\dot{\theta }}_{4}m_{LF}s_{234}-2L_{S}{\dot{\theta }}_{2}{\dot{\theta }}_{3}m_{LF}s_{23}-L_{S}{\dot{\theta }}_{2}{\dot{\theta }}_{3}m_{LS}s_{23}-R_{F}{\dot{\theta }}_{5}{\dot{\theta }}_{6}m_{RF}s_{567}-R_{F}{\dot{\theta }}_{5}{\dot{\theta }}_{7}m_{RF}s_{567}\\ \;-R_{F}{\dot{\theta }}_{6}{\dot{\theta }}_{7}m_{RF}s_{567}-2R_{S}{\dot{\theta }}_{5}{\dot{\theta }}_{6}m_{RF}s_{56}-R_{S}{\dot{\theta }}_{5}{\dot{\theta }}_{6}m_{RS}s_{56}\\ G_{1}=\frac{L_{body}gm_{body}\cos\theta_{1}}{2}\\ G_{2}=\frac{g\left(L_{F}c_{234}m_{LF}+2L_{S}c_{23}m_{LF}+L_{S}c_{23}m_{LS}+2L_{T}c_{2}m_{LF}+2L_{T}c_{2}m_{LS}+L_{T}c_{2}m_{LT}\right)}{2}\\ G_{3}=\frac{g\left(L_{F}c_{234}m_{LF}+2L_{S}c_{23}m_{LF}+L_{S}c_{23}m_{LS}\right)}{2}\\ G_{4}=\frac{L_{F}c_{234}gm_{LF}}{2}\\ G_{5}=\frac{g\left(R_{F}c_{567}m_{RF}+2R_{S}c_{56}m_{RF}+R_{S}c_{56}m_{RS}+2R_{T}c_{5}m_{RF}+2R_{T}c_{5}m_{RS}+R_{T}c_{5}m_{RT}\right)}{2}\\ G_{6}=\frac{g\left(R_{F}c_{567}m_{RF}+2R_{S}c_{56}m_{RF}+R_{S}c_{56}m_{RS}\right)}{2}\\ G_{7}=\frac{R_{F}c_{567}gm_{RF}}{2}\\ G_{8}=0\\ G_{9}=g\left(m_{LF}+m_{LS}+m_{LT}+m_{RF}+m_{RS}+m_{RT}+m_{body}\right) \end{array}$$
